# Entinostat in patients with relapsed or refractory abdominal neuroendocrine tumors

**DOI:** 10.1093/oncolo/oyae118

**Published:** 2024-05-31

**Authors:** Jacob K Jamison, Mengxi Zhou, Edward P Gelmann, Lyndon Luk, Susan E Bates, Andrea Califano, Tito Fojo

**Affiliations:** Weill Cornell Medical College, New York, NY, United States; Department of Medicine, Division of Hematology Oncology, Columbia University Irving Medical Center, New York, NY, United States; University of Arizona Cancer Center, Tucson, AZ, United States; Department of Radiology, Columbia University Irving Medical Center, New York, NY, United States; Department of Medicine, Division of Hematology Oncology, Columbia University Irving Medical Center, New York, NY, United States; Department of Medicine, Division of Hematology Oncology, Columbia University Irving Medical Center, New York, NY, United States; Department of Systems Biology, Columbia University Irving Medical Center, New York, NY, United States; Department of Biochemistry and Molecular Biophysics, Columbia University Irving Medical Center, New York, NY, United States; Chan Zuckerberg Biohub New York, New York, NY, United States; Department of Biomedical Informatics, Columbia University Irving Medical Center, New York, United States; Herbert Irving Comprehensive Cancer Center, Columbia University Irving Medical Center, New York, United States; Department of Medicine, Division of Hematology Oncology, Columbia University Irving Medical Center, New York, NY, United States

**Keywords:** neuroendocrine tumor, entinostat, GEP-NET, HDAC inhibitors, tumor growth rate

## Abstract

**Background:**

Gastroenteropancreatic neuroendocrine tumors (GEP-NETs) are rare neoplasms with an increasing annual incidence and prevalence. Many are metastatic at presentation or recur following surgical resection and require systemic therapy, for which somatostatin analogs such as octreotide or lanreotide comprise typical first-line therapies. Nonetheless, treatment options remain limited. Epigenetic processes such as histone modifications have been implicated in malignant transformation and progression. In this study, we evaluated the anti-proliferative effects of a histone deacetylase (HDAC) inhibitor, entinostat, which was computationally predicted to show anti-cancer activity, as confirmed in in vitro and in vivo models of GEP-NETs.

**Methods:**

This was a phase II study to evaluate the efficacy and safety of entinostat in patients with relapsed or refractory abdominal NETs. The primary objective was to estimate the objective response rate to entinostat. Additionally, with each patient as his/her own control we estimated the rates of tumor growth prior to enrollment on study and while receiving entinostat. Patients received 5 mg entinostat weekly until disease progression or intolerable toxicity. The dose could be changed to 10 mg biweekly for patients who did not experience grade ≥ 2 treatment-related adverse events (AEs) in cycle 1, but was primarily administered at the starting 5 mg weekly dose.

**Results:**

The study enrolled only 5 patients due to early termination by the drug sponsor. The first patient that enrolled had advanced disease and died within days of enrollment before follow-up imaging due to a grade 5 AE unrelated to study treatment and was considered non-evaluable. Best RECIST response for the remaining 4 patients was stable disease (SD) with time on study of 154+, 243, 574, and 741 days. With each patient as his/her own control, rates of tumor growth on entinostat were markedly reduced with rates 17%, 20%, 33%, and 68% of the rates prior to enrollment on study. Toxicities possibly or definitely related to entinostat included grade 2/3 neutrophil count decrease [2/4 (50%)/ 2/4 (50%)], grade 3 hypophosphatemia [1/4, (25%)], grade 1/2 fatigue [1/4 (25%)/ 2/4 (50%)], and other self-limiting grade 1/2 AEs.

**Conclusion:**

In the treatment of relapsed or refractory abdominal NETs, entinostat 5 mg weekly led to prolonged SD and reduced the rate of tumor growth by 32% to 83% with an acceptable safety profile (ClinicalTrials.gov Identifier: NCT03211988).

## Teaser

Treatment options for gastroenteropancreatic neuroendocrine tumors (GEP-NETs) are limited. This study evaluated the anti-proliferative effects of a HDAC inhibitor, entinostat, which has previously shown anti-cancer activity in in vitro and in vivo models of GEP-NETs.

**Lessons learned**

- In patients with relapsed/refractory abdominal neuroendocrine tumors, the histone deacetylase inhibitor, entinostat, was well tolerated and reduced rates of tumor growth by 32%-80% with tumor doubling times of 297, 724, 1149, and 2214 days.
- Conducted during the pandemic weekly administration as outpatients provided a convenient treatment platform.
- A novel method of efficacy analysis that includes time in equations that estimate tumor growth rates allowed each patient to serve as his/her own control—valuable for small trials.

## Discussion

Neuroendocrine tumors (NETs) are derived from neuroendocrine cells and include a heterogenous group of rare neoplasms that often affect organs of the gastroenteropancreatic (GEP) tract.^1^ These tumors are somewhat unique due to the release of bioactive amines and peptides such as gastrin or insulin. Up to 27%^1^ of NETs present with metastatic disease, and up to 42%^2^ recur following surgical resection, such that systemic therapy is needed. Somatostatin analogs such as octreotide or lanreotide have shown significant prolongation of progression-free survival among patients with metastatic GEP-NETs and typically constitute first-line therapies.^3^

We studied the histone deacetylase inhibitor (HDAC) entinostat in patients with neuroendocrine tumors. The study was based on preclinical data demonstrating entinostat-mediated, *in vivo* inversion of master regulator protein activity signatures, as predicted by the VIPER-based OncoTreat algorithm.^4^^5^ These effects are consistent with our understanding of HDAC inhibition allowing hyperacetylation, open chromatin and induction of genes that control cell growth and differentiation. Other lines of evidence suggesting particular sensitivity of neuroendocrine cancers to HDAC inhibitors have been gathered including both clinical and preclinical studies.^6^

The present study examining entinostat given as a 5 mg oral dose every week or 10 mg every other week in patients with relapsed or refractory abdominal NETs showed an acceptable safety profile with no dose-limiting toxicity. Side effects were consistent with those previously reported including fatigue, neutropenia, and hypophosphatemia (Table 1). All evaluable patients achieved best response of stable disease per RECIST, with 3 of 4 maintaining this response at study closure, resulting in a median progression-free survival of 381 + days. Use of a novel method of efficacy analysis that allowed each patient to serve as his/her own control demonstrated a reduction in the rate of tumor growth (ie, an increase in tumor doubling time) in all patients. Although the study was prematurely closed due to sponsor decision regarding entire drug development plan, these data support further investigation of HDAC inhibitors in this patient population.

**Table 1.** Adverse events.

Event	Grade 1	Grade 2	Grade 3	Grade 4
Abdominal pain	1			
Anemia	1			
Anorexia	1			
Bloating	1			
Diarrhea	1			
Fatigue	1	2		
Fever	2			
Flu-like symptoms	1			
Nausea	3			
Night sweats	1			
Vomiting	1			
Neutrophil count decreased		2	2	
Platelet count decreased	1			
Hypophosphatemia			1	

**Table 2.** Summary data for patients.

	Patient 2	Patient 3	Patient 4	Patient 5
Date of diagnosis	07/25/2017	05/19/2017	03/04/2019	12/31/2012
Date of enrollment/C1D1	8/14/2019	8/13/2019	2/12/2020	10/27/2020
Age at enrollment	66 years	59 years	77 years	82 years
Primary tumor location	Small bowel (resected)	Pancreatic tail	Pancreatic body	Pancreatic tail (resected)
Diagnosis method	Liver core biopsy	Liver core biopsy	Pancreas and liver EUS-guided core biopsies	Distal pancreatectomy
Differentiation	Well differentiated	Poorly differentiated	Well differentiated	Well differentiated
Ki67 Index	1%-2%	5%	4%	10%
Prior treatment (duration)	Surgical resection of small bowel Lanreotide (7 mo)	Lanreotide (2 yr)	Lanreotide (7 mo) Octreotide (2 mo)	Distal pancreatectomy Lanreotide (7 mo) Octreotide (12 mo)
Pre-enrollment gallium-DOTATATE avidity	Liver Abdominal lymph nodes	Pancreatic tail Liver Portacaval and peripancreatic lymph nodes	Pancreatic body Liver Bone (left 3rd rib)	Liver Peritoneum Left adrenal gland vs lymph node
Pre-enrollment chromogranin-A (ng/mL, months prior to enrollment)	92 (<1)	61 (<1)	171 (1)	210 (10)
Time on study	24 months	18 months	8 months	5 months
Best overall response per RECIST	−20%	0%	0%	−10%

## Trial Information

**Table:** 

Disease	Relapsed or refractory abdominal neuroendocrine tumors
Stage of disease/treatment	IV
Prior therapy	≥1 prior regimen
Type of study	Phase II
Primary endpoint	Objective RECIST response based on patient’s best response that is documented during protocol therapy
Secondary endpoints	Duration of response, overall survival, progression-free survival, and safety

## Additional details of endpoints or study design

### Mathematical model

The model assumes that changes in tumor quantity during treatment result from two processes—exponential decay of the treatment-sensitive fraction of tumor at rate *d*, and exponential growth or re-growth of the resistant fraction at rate *g* (Figure 2). The data of most tumors can be fitted to 1 of 4 equations:


$$\begin{array}{l} f(t)=exp(-d•t)+exp(g•t)-1 \end{array}$$ (1)

$$\begin{array}{l} f(t)=exp(g•t) \end{array}$$ (2)

$$\begin{array}{l} f(t)=exp(-d•t) \end{array}$$ (3)

$$\begin{array}{l} f(t)=\Phi exp(-d•t)+(1-\Phi )exp(g•t) \end{array}$$ (4)

where *f*(*t*) represents tumor burden at time *t* (in days), relative to a quantity of 1 for radiographic measurements at *t* = 0. *g* (in days^−1^) is the rate of growth and is related to tumor doubling time (*T*_*d*_) by the formula *T*_*d*_* *= 0.693/*g*, where 0.693 is the natural logarithm of 2. *d* (in days^−1^) is the rate of decay. φ is the treatment-sensitive fraction of tumor and (1–φ) is the fraction absolutely or relatively resistant to treatment.

The basic model *gd* is described in eqn (1). In cases where the data demonstrate only an increase in tumor burden from the beginning of therapy (ie, only *g* differs significantly from 0 with *P* < .1), *d* is eliminated, and tumor *g*rowth rate is estimated using the *gx* equation (eqn (2)). Similarly, in cases where the data demonstrate only a reduction in tumor burden from the beginning of therapy (ie, only *d* differs significantly from 0 with *P* < .1), *g* is eliminated, and tumor decay rate is estimated using the *dx* equation (eqn (3)). ϕ represents the proportion of tumor that is sensitive to therapy. In cases where the data allow the estimation of 3 parameters, ϕ can be estimated using the *gdϕ* equation (eqn (4)). Incorporation of time (*t*) in the equations renders the analysis indifferent to time (ie, intervals of assessment used by the study).

## Drug Information

**Table:** 

Generic/working name	Entinostat
Company name	Syndax
Drug type	Small molecule
Drug class	HDAC inhibitor
Dose	5 mg
Unit	N/A
Route	PO
Schedule of administration	Once weekly

## Patient Characteristics

**Table:** 

Number of patients, male	4
Number of patients, female	1
Stage	IV
Age: median (range)	75 (59-82) years
Number of prior systemic therapies: median (range)	1.5 (1-2)
Performance status: ECOG	1: 4 2: 0 3: 0 4: 0 5: 0
Cancer types or histologic subtypes	Small bowel NET, 2; pancreatic NET, 2

## Primary Assessment Method: Clinical Response

**Table:** 

Number of patients screened	5
Number of patients enrolled	5
Number of patients evaluable for toxicity	4
Number of patients evaluated for efficacy	4
Evaluation method	RECIST 1.1
Response assessment, SD	4 (100%)
Median duration assessments, PFS	381 + days (232+, 253+, 510+, 754)
Median duration assessments, OS	1,422 + days (1163+, 1240, 1603+, 1611+)
Duration of treatment	409 days (154+, 243, 574, and 741)
Outcome notes	With each patient as his/her own control, rates of tumor growth on entinostat were 20, 33, 54, and 68 percent of rates prior to enrollment on study, with tumor doubling times of 297, 724, 1149, and 2214 days (Figure 1)

**Figure 1.**
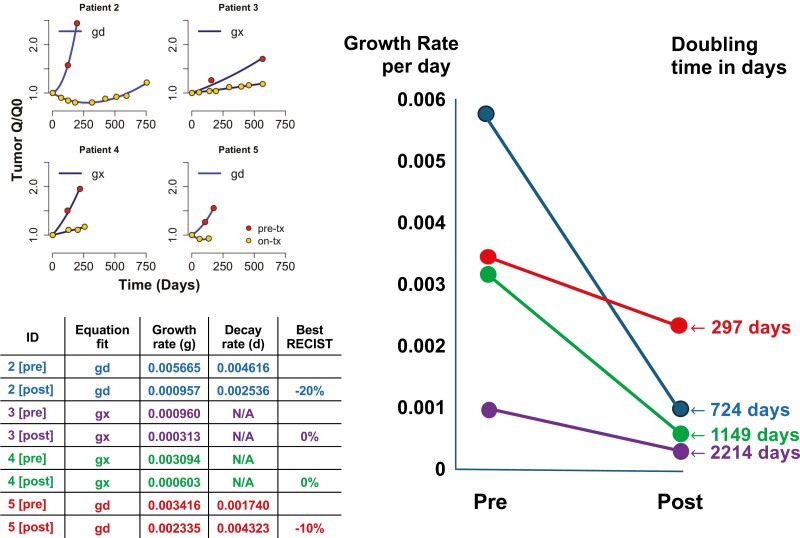
Tumor growth rates.

## Assessment, Analysis, and Discussion

**Table:** 

Completion	Study terminated prior to completion
Investigator’s assessment	Active and should be pursued further

Neuroendocrine tumors (NETs) are derived from neuroendocrine cells and include a heterogenous group of rare neoplasms that often affect organs of the GEP tract.^1^ Associated symptoms depend on the location of disease and are caused by mass effect or release of biogenic amines and peptides.^2^ Multiple studies have reported an increase in the incidence and prevalence of NETs in the US, the largest of which used the Surveillance, Epidemiology and End Result registry to report a 6-fold age-adjusted incidence rate increase from 1.09 to 6.98 per 100 000 between 1973 and 2012.^1^ Up to 27%^1^ of NETs (and as many as 40%^3^ of GEP-NETs) present with metastatic disease, and up to 42%^5^ recur following surgical resection, thus many patients require systemic therapy during the course of their disease. Somatostatin analogs such as octreotide or lanreotide have shown significant prolongation of progression-free survival among patients with metastatic GEP-NETs and typically constitute first-line therapies.^6,7^

Among mechanisms implicated in cancer transformation and progression, epigenetic changes can alter gene expression without alteration of the DNA sequence.^8^ Multiple post-translational modifications on amino acid residues have been described affecting the 5ʹ region of histone proteins that form the octamer around which DNA is wrapped. One of the most widely studied is histone acetylation. According to the canonical mechanism, post-translational acetylation of lysine residues in histone “tails” impairs the electrostatic interaction between histones and DNA, leading to DNA relaxation and increased gene transcription. Two families of enzymes—the histone acetyltransferases (HATs) and histone deacetylases (HDACs)—modulate epigenetic histone acetylation.^9^ Alterations in the balance between HATs and HDACs can lead to changes in gene expression that in turn affect cell proliferation and survival and have made these enzymes attractive targets for cancer therapy.^10^

Entinostat, also known as MS275 or SNDX-275, is an orally available synthetic pyridylcarbamate HDAC inhibitor that has demonstrated antitumor efficacy in vitro and in vivo through multiple proposed mechanisms including activity as an HDAC inhibitor leading to hyperacetylation of nucleosomal histones. Treatment of cancer cell lines with entinostat at concentrations ranging from 0.0415 to 4.71 μM resulted in accumulation of hyperacetylated histones and an antiproliferative effect in a variety of models including leukemia (K562, HL-60), ovarian cancer (A2780), and colorectal cancer (HCT-15).^11^ Similar results have been found in vivo for a wide spectrum of cancer xenograft models including melanoma, prostate, breast, NSCLC, pancreas, colon, ovarian, gastric, head and neck, and glioblastoma.^12^ Entinostat has also been shown to modify the phenotype of breast cancer cells from a mesenchymal to an epithelial one, with a corresponding reduction in metastatic potential.^13^

Pre-clinical data suggest that compared to other HDAC inhibitors, entinostat may have greater efficacy in the treatment of GEP-NETs. For example, entinostat led to greater in vivo tumor growth inhibition (112%) when tested in the H-STS GEP-NET xenograft model compared to the pan-HDAC inhibitor belinostat (8%).^4^ A major difference between entinostat and other HDAC inhibitors is its pure class I HDAC activity. The lack of acetylation of proteins in the cytoplasm may mean that at therapeutic doses the drug has a mainly epigenetic effect. Consistent with this, entinostat’s efficacy was predicted based on its ability to inhibit the activity of master regulator proteins representing mechanistic determinants of the transcriptional state of GEP-NET liver metastasis cells.^4^ While we understand “epigenetic” to mean alteration in gene expression, other effects of histone hyperacetylation may also be invoked. There may also be an important role for histone hyperacetylation resulting in depletion of acetyl CoA thereby inducing metabolic vulnerability, and stress^14^ which may be in part the basis of synergy observed in combination with NAMPT inhibitors.^15^ Additionally, persistently open chromatin appears to lead to DNA damage with the formation of RNA-DNA hybrids during gene transcription.^16^

Support for entinostat as a safe and effective therapeutic approach includes the FDA approvals of other HDAC inhibitors in the treatment of T-cell lymphomas.^17^ It also includes data from the large randomized trial of entinostat and exemestane in breast cancer that, while not showing efficacy beyond exemestane alone, confirmed safety of the agent in 608 patients.^18^ Additionally, early phase studies of entinostat in solid tumors such as non-small cell lung cancer have demonstrated tolerability at doses up to 10 mg, with the most commonly reported adverse events (AEs) including fatigue and gastrointestinal disturbances.^19^ A phase I study with belinostat, cisplatin, and etoposide that focused on small cell lung cancer and neuroendocrine cancers showed an objective response in 7 of 15 (47%) patients, suggesting that the combination should be pursued further.^20^

The present study examining entinostat given as a 5 mg oral dose every week or 10 mg every other week in patients with relapsed or refractory abdominal NETs showed an acceptable safety profile with no dose-limiting toxicity. Grade 2/3 adverse events possibly or definitely related to entinostat were consistent with prior clinical studies of its use in solid tumors and included fatigue, neutropenia, and hypophosphatemia (Table 1).^19,21^ All evaluable patients achieved best response of stable disease per RECIST, with 3 of 4 maintaining this response at study closure, resulting in a median progression-free survival of 381 + days. Use of a novel method of efficacy analysis that allowed each patient to serve as his/her own control demonstrated a reduction in the rate of tumor growth (ie, an increase in tumor doubling time) in all patients and may serve as a valuable metric of drug activity for small trials (Figure 1). A best RECIST tumor response of −20% was achieved in a patient with a small bowel NET metastatic to the liver previously treated with surgical resection and 7 months of lanreotide (Table 2). Entinostat’s ability to achieve clinical activity in a cohort of pre-treated patients suggests that further study is warranted, particularly with regard to longer-term outcomes. Additionally, its oral route of intake provided a convenient treatment option for patients during the COVID-19 pandemic. Unfortunately, the sponsor deprioritized development of entinostat, and slow patient accrual during the pandemic made more sophisticated analyses impractical due to lack of statistical power. In summary, further study of entinostat and other HDAC inhibitors in treatment-refractory GEP-NETs is warranted.

**Figure 2.**
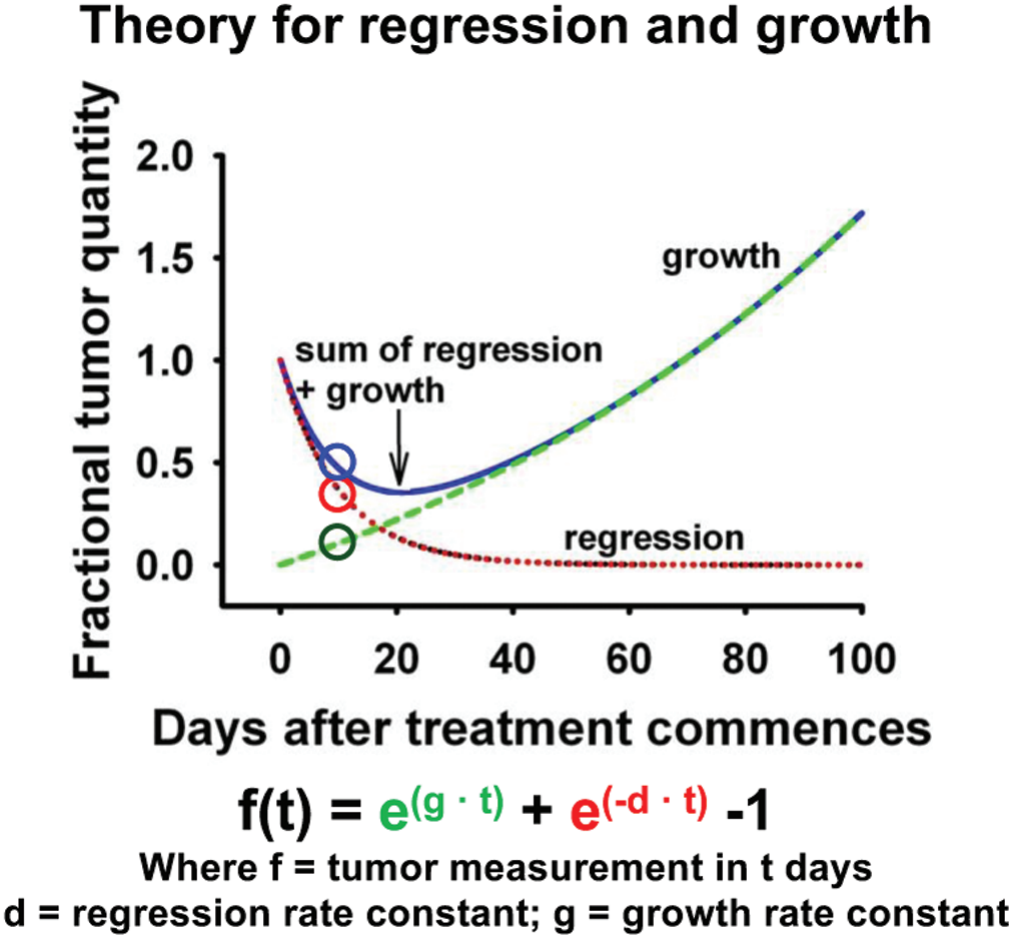
Theory for regression and growth.

## Data Availability

The data underlying this article cannot be shared publicly due to patient confidentiality. The data will be shared on reasonable request to the corresponding author.
