# Correction to: Entinostat in patients with relapsed or refractory abdominal neuroendocrine tumors

**DOI:** 10.1093/oncolo/oyae199

**Published:** 2024-08-31

This is a correction to: Jacob K Jamison, Mengxi Zhou, Edward P Gelmann, Lyndon Luk, Susan E Bates, Andrea Califano, Tito Fojo, Entinostat in patients with relapsed or refractory abdominal neuroendocrine tumors, The Oncologist, 2024; https://doi.org/10.1093/oncolo/oyae118

The following changes have been made to the originally published manuscript.

Mark-up ‡ has been added to author names Andrea Califano and Tito Fojo in the author byeline and statement added to indicate their equal contribution.

The credential “Dr” was added for name Andrea Califano in the statement re corresponding authors.

In section **Abstract**, under **Results**, text in 4th sentence was changed to read: “[…]rates of tumor growth on entinostat were markedly reduced with rates 17%, 20%, 33%, and 68%[…]” instead of: “[..]rates of tumor growth on entinostat were markedly reduced with rates 20%, 33%, 54%, and 68%[…]”.

In section **Abstract**,under Conclusion, text in the first sentence was changed to read: “[…]reduced the rate of tumor growth by 32% to 83%[…]” instead of: ““[…]reduced the rate of tumor growth by 32% to 80%[…]”.

In section **Lessons Learned**, text in the first point, was changed to read: “[…]reduced the rate of tumor growth by 32% to 83%,[…]” instead of: ““[…]reduced the rate of tumor growth by 32% to 80%,[…]”.

In section **Primary Assessment Method: Clinical Response**, line **Outcome notes** was changed to read: “[…]rates of tumor growth on entinostat were 17, 20, 33, and 68 percent of rates[…]” instead of: ““[…]rates of tumor growth on entinostat were 20, 33, 54, and 68 percent of rates”.

In section Assessment, Analysis and Discussion, in the sixth paragraph, references and links to tables were emended as follows: the end of the second sentence, to read: “[...]hypophosphatemia (Table 1).” instead of: “[...]hypophosphatemia (Table 2).”; the end of the fourth sentence to read: “[...] for small trials (Figure 1).” instead of: “[...] for small trials (Figure 2).”; and the end of the fifth sentence to read: “[...] lanreotide (Table 2).” instead of: “[...] lanreotide.”

The emendations have been made to the article

